# Differences in the Determination of Volatile Organic Compounds between *Chrysanthemum morifolium* Ramat. and *Chrysanthemum indicum* L. (Wild Chrysanthemum) by HS-GC-IMS

**DOI:** 10.3390/molecules29194609

**Published:** 2024-09-27

**Authors:** Gaigai Liu, Hao Duan, Yue Zheng, Jinhong Guo, Diandian Wang, Wenjie Yan

**Affiliations:** 1 College of Biochemical Engineering, Beijing Union University, Beijing 100023, China; lgg010403@163.com (G.L.); dhuanao@163.com (H.D.); zheng_yue21@163.com (Y.Z.); gjh121133@163.com (J.G.); spwdd2018@163.com (D.W.); 2 Beijing Key Laboratory of Bioactive Substances and Functional Food, Beijing Union University, Beijing 100023, China; 3 College of Food Science and Engineering, Northwest A&F University, 22 Xinong Road, Yangling, Xianyang 712100, China

**Keywords:** chrysanthemum, wild chrysanthemum, HS-GC-IMS, volatile organic compound

## Abstract

Chrysanthemums and wild chrysanthemums are herbs with high application value. As edible plants of the Asteraceae family, they have good antioxidant, anti-inflammatory and hepatoprotective properties. Chrysanthemums and wild chrysanthemums contain a wide variety of volatile organic compounds, and these volatile components are the main factors contributing to the flavor differences. Therefore, in this study, we investigated the volatile components of holland chrysanthemum from Bozhou, Anhui Province, Chu-chrysanthemum from Chuzhou, Anhui Province, Gong-chrysanthemums from Huangshan, Anhui Province, Huai-chrysanthemums from Jiaozuo, Henan Province, Hang-chrysanthemum from Hangzhou, Zhejiang Province, and wild chrysanthemum from Dabie Mountain by headspace–gas chromatography–ion mobility spectrometry (HS-GC-IMS) coupled with principal component analysis (PCA). The results showed that Chrysanthemum and wild chrysanthemum contain alcohols, esters, hydrocarbons, ketones, aldehydes, acids, camphor, pyrazines and furans. Among them, alcohols, esters and hydrocarbons accounted for more than 15%. It was hypothesized that 2-methyl-1-propanol, 2-methylbutanol, 1-hexanol in alcohols and hexyl acetate, 3-methylbutyl acetate and ethyl 2-methylpropanoate in esters might be the main reasons for the alcoholic and sweet flavors of chrysanthemum and chrysanthemum officinale. Based on the principal component analysis, cluster analysis with the Euclidean distance and similarity analysis of fingerprints, it was found that there were significant differences in the volatile components in chrysanthemums from different origins, among which the differences between Chu-chrysanthemum and Hang-chrysanthemum were the most significant. In addition, as a genus of wild chrysanthemum with the same species, it contains a richer variety of volatile organic compounds, and the content of hydrocarbons and alcohols is significantly higher than that of chrysanthemum.

## 1. Introduction

Chrysanthemums (*Chrysanthemum morifolium Ramat.*) and wild chrysanthemums (*Chrysanthemum indicum* L.) are perennial herbaceous plants belonging to the family Asteraceae, of which chrysanthemums are considered to be one of the most economically valuable edible flowers in the world [1]. Chrysanthemum is distributed in China, Japan, Korea and Russia and other Asian countries, and even in the Netherlands and other European countries have some distribution [2]. Wild chrysanthemum is more widely distributed, from grassland, plains to hills. At present, China is the world’s largest producer of chrysanthemum [3]. In China, chrysanthemum plantations are mainly concentrated in Henan, Anhui, Hubei and Zhejiang. In the Chinese Pharmacopoeia of the People’s Republic of China (2020 edition), chrysanthemums are mainly mentioned as Bo-chrysanthemum from Bozhou, Anhui; Chu-chrysanthemum from Chuzhou, Anhui; Gong-chrysanthemum from Huangshan, Anhui; Huai-chrysanthemum from Jiaozuo, Henan; and Hang-chrysanthemum from Hangzhou, Zhejiang, as well as [4]. Modern research has confirmed that chrysanthemum and wild chrysanthemum have significant effects in lowering blood pressure, blood lipids and blood glucose, and also have the effect of clearing the liver and brightening the eyes [5]. They can not only be used in medicine, in which chrysanthemum also has a wide range of applications in health care functional food, known as the most representative flower-derived dietary herbs [6]. However, chrysanthemums and wild chrysanthemums from different origins have significant differences in composition and pharmacological activity [7].

The current analysis of chrysanthemum-related flavors mainly focuses on ornamental chrysanthemums, and little research has been conducted on edible chrysanthemums, especially for those mentioned in the Pharmacopoeia of the People’s Republic of China (2020 edition) [4], which were not analyzed and compared in more detail. A literature search revealed that chrysanthemums from their origins are more widely used and studied [8]. Therefore, chrysanthemums and wild chrysanthemums from six different origins were analyzed for more in-depth flavor substances.

Odor is one of the main factors in identifying the quality of herbs when used as marketed or functional foods, and the volatile flavor components they contain play an important role in the acceptability of chrysanthemums and wild chrysanthemums. Chrysanthemum is usually “sweet and bitter”, while wild chrysanthemum is “aromatic and bitter” [4]. Therefore, the comparison of the volatile components of chrysanthemums and wild chrysanthemums from different origins deserves in-depth study.

Currently, flavor components are generally analyzed by sensory and instrumental techniques [9]. One is to utilize sensory perception for subjective evaluation, and to evaluate the samples accordingly by providing certain training to the relevant personnel [10]. However, it has uncertainty and can be interfered by various disturbing factors, such as the environment, the rater’s mood, and physical condition [11]. The other is the use of instruments, such as electronic noses and gas chromatography, which are more objective than subjective evaluations and have a relatively high degree of accuracy. Among them, electronic noses are used for real-time monitoring of odor fingerprints and their changes in the samples by means of electronic sensors, but electronic noses identify the compounds as a whole and cannot analyze the single components [12,13]. Chromatographic techniques, on the other hand, can qualitatively and quantitatively analyze the samples by detecting the structure of the chemical components and combining them with their relative molecular masses, which can obtain the results in a short period of time, and solve the disadvantage of the electronic nose with slightly lower reproducibility [14,15,16].

In recent years, chromatographic techniques have been improved, mainly liquid chromatography–mass spectrometry (HPLC-MS), gas chromatography–mass spectrometry (GC-MS), gas chromatography–ion mobility spectrometry (GC-IMS) and headspace–gas chromatography–ion mobility spectrometry (HS-GC-IMS) [17,18]. For the analysis of volatile and semi-volatile substances, the headspace–gas chromatography–ion mobility spectrometry (HS-GC-IMS) technique is used. This technique is not only easy to operate, but also fast, sensitive and has a higher separation efficiency than GC-MS [19]. For example, the identification of volatile components of chamomile, chrysanthemum, and chrysanthemum tea was carried out using this technique, and a total of 47 chemical substances were identified [20]. Currently, analyses of the volatile constituents of chrysanthemums have focused on related tea broths and ornamental chrysanthemums [8,21], and there has been no identification and comparison of volatile constituents between chrysanthemums and wild chrysanthemums in the Pharmacopoeia of the People’s Republic of China (2020 edition).

This study utilized a combination of HS-GC-IMS and PCA, hierarchical cluster analysis heat map to efficiently and intuitively identify and classify the volatiles in chrysanthemums and wild chrysanthemums, and more volatile organic compounds were found. In the future, in the application process of related products, we can keep the ingredients with special aroma that do not have significant irritating odor, and we can also reduce the effect of the ingredients causing irritating odor on the products by certain technical means. We hope that the present study can provide a certain theoretical basis and application value for the future screening of raw materials of chrysanthemum and wild chrysanthemum and the enhancement of the nutritional value of functional food products.

## 2. Results

### 2.1. HS-GC-IMS Topography of Chrysanthemum and Wild Chrysanthemum Samples from Different Origins

**Figure 1:**
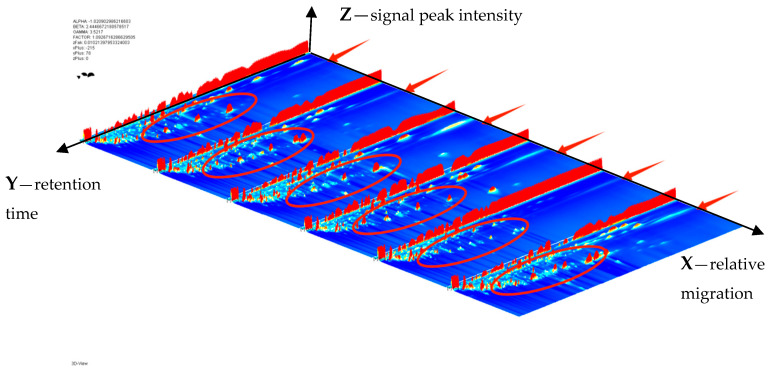
3D geomorphological map.

In this study, HS-GC-IMS was used to analyze the differences in volatile organic compounds in chrysanthemums and wild chrysanthemums from different origins. The generated data were represented as 3D spectrograms with three axes representing relative migration time (*X*-axis), retention time (*Y*-axis) and signal peak intensity (*Z*-axis). As can be seen in Figure 1, the volatile compounds in chrysanthemums and wild chrysanthemums from different origins are very similar, but there are some differences, and it can be seen that the signal intensities shown in the red circles are slightly different.

**Figure 2:**
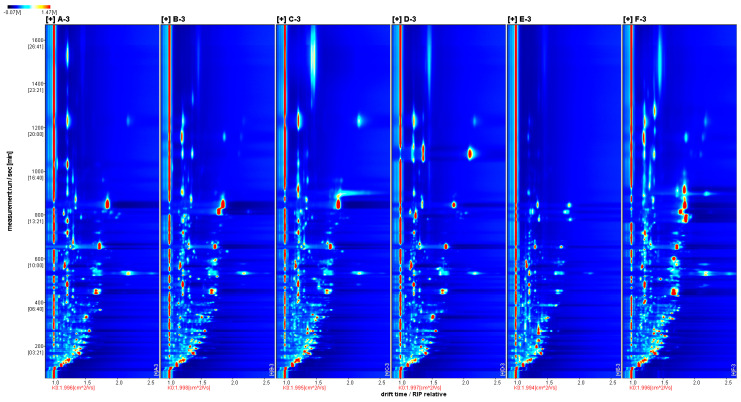
Topographic maps of all samples. (A: Huai-chrysanthemum; B: Bo-chrysanthemum; C: Chu-chrysanthemum; D: Gong-chrysanthemum; E: Hang-chrysanthemum; F: Wild chrysanthemum cm^2^).

In order to obtain a better view of these differences, these samples were compared in more detail using a top view. As shown in Figure 2, the blue color was chosen as the background color of the whole graph, and the red vertical line at the horizontal coordinate 1.0 was the RIP peak (reactive ion peak, normalized). The vertical coordinate represents the retention time (s) of the GC and the horizontal coordinate represents the relative migration time (normalized). Each point on either side of the RIP peak represents a VOC. The color represents the intensity of a substance’s peak, from blue to red, with darker colors indicating greater peak intensity. From the figure, it can be seen that there are some differences in the VOCs in different sample samples.

**Figure 3:**
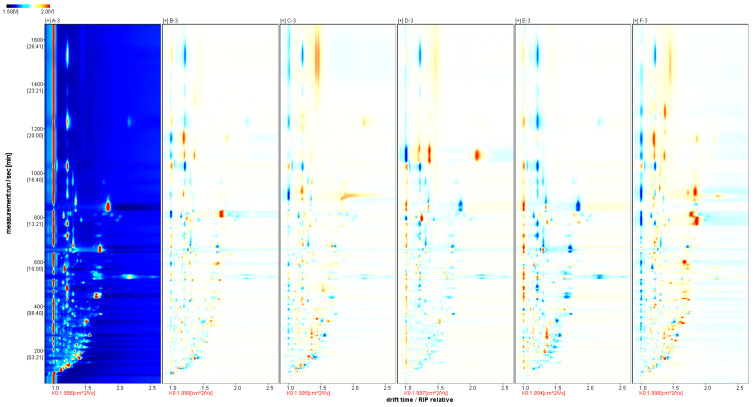
GC-IMS difference spectrum of volatile components.

Although the topographic maps can show the differences in volatile compositions of chrysanthemum and wild chrysanthemum samples from different origins, it is not possible to visualize the differences in a more significant way. The spectrum of sample A was selected as the reference, and the spectra of other samples were deducted from the reference to obtain the difference comparison graph of different samples, as shown in Figure 3. If the VOC content is the same in the target sample and the reference, the background of the deduction is white, while the red color indicates that the concentration of the substance is higher in the target sample than in the reference, and the blue color indicates that the concentration of the substance is lower in the red color of the target sample than in the reference.

Comparing Figure 2 and Figure 3, it can be found that most of the signals have retention times of 50–1350 s and drift times of 1.0–2.3 ms. Moreover, in the differential contrast model plot (Figure 3), the concentration of different volatile components can be seen. Moreover, in the differential contrast model plot (Figure 3), the concentration of different volatile components can be seen. The retention times were 650–850 s and the drift times were 1.6–1.9 ms for all the samples except sample D. The darker the red color in the graph, the stronger the signal strength of the substance. The content of 4-isopropyltoluene in sample D was significantly higher than that in other samples at retention times of 1050–1150 s and drift times of 2.1–2.3 ms; the content of camphoraceous substances in sample A was significantly higher than that in other samples at retention times of 650–850 s and drift times of 1.7–1.9 ms; the content of (E, E)-2,4-octadienal was significantly higher than that in other samples at retention times of 750–850 s and drift times of 1.6–1.8 ms. The content of (E, E)-2,4-octadienal was significantly higher than that of other samples at the retention time of 750–850 s and drift time of 1.6–1.8 ms. The analysis showed that the concentration of volatile organic compounds (VOCs) contained in sample D was higher than that of samples A, B, C and E, indicating that Gong-chrysanthemum had a higher content of volatile components in these five chrysanthemum samples.

### 2.2. Comparative Analysis of the Fingerprints of Volatile Components in Chrysanthemum and Wild Chrysanthemum Samples from Different Origins

**Figure 4:**
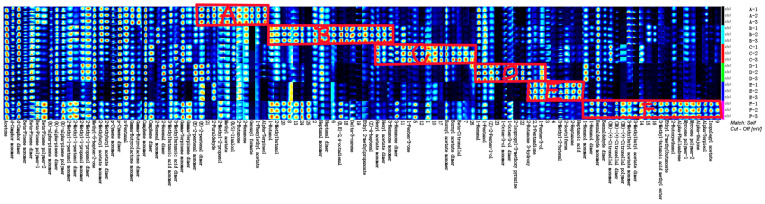
Fingerprints of volatile components. (A: Huai-chrysanthemum; B: Bo-chrysanthemum; C: Chu-chrysanthemum; D: Gong-chrysanthemum; E: Hang-chrysanthemum; F: Wild chrysanthemum cm^2^).

Fingerprinting allows a complete and clear comparison of the differences in specific volatile substances in chrysanthemums and wild chrysanthemums of different origins. As shown in Figure 4, each row of the graph represents all the signal peaks selected in a sample, and each column represents the signal peaks of the same VOC in different samples, and the color shades represent the signal strengths, and the darker the color indicates that the signal strength of the substance is stronger, and its content is higher.

The complete VOC information for each sample and the differences in VOCs between samples can be seen in Figure 4. Samples D and E had fewer species and concentrations, while sample F had the most volatiles. Wild chrysanthemum has the largest variety and content of volatiles, indicating a more distinctive flavor profile. The volatiles in the six different samples were further compared, and the fingerprints of all volatiles were analyzed, as shown in Figure 4. The results of the comparative analysis showed that the differences in the volatile substances of the six chrysanthemums were large.

2-Methyl-2-propanol, linalool, 2-butanone, 2-hexanone, formic acid geranyl ester, 1-phenylethyl acetate, and alpha-pinacol were the characteristic substances of sample A. heptanal, (2E,4E)-2,4-octadienal, 3-carene, ethyl 2-methylpropionate, Z-4-heptenal, and hexyl acetate are characteristic substances of sample B. 3-Hexanone, 1-penten-3-one, bornyl acetate, citronellal are characteristic substances of sample C. Hexanal, pentanol, 1-octen-3-ol, 2-isopropyl-3-methoxypyrazine are the characteristic substances of sample D.Acetoin,2,3-pentanedione,1-penten-3-ol,3-methyl-2-butenal,2-acetylfuran,2-heptanone, and heptanoic acid are the characteristic substances of sample E. Hexanol, benzaldehyde, citronellal, isoamyl acetate, methyl 3-methylbutyrate, ethyl 2-methylbutyrate ether are characteristic substances of sample F. Chrysanthemums and wild chrysanthemums of different origins have characteristic volatile organic compounds, mainly alcohols, aldehydes, esters and ketones. Among them, the volatiles of chrysanthemum and Gong-chrysanthemum were mainly alcohols, the volatiles of chrysanthemum were mainly aldehydes, the volatiles of Chu-chrysanthemum and Hang-chrysanthemum were mainly ketones, and the volatiles of wild chrysanthemum were mainly esters.

### 2.3. Cluster Analysis of Volatile Components of Chrysanthemum and Wild Chrysanthemum Samples from Different Origins

Principal Component Analysis (PCA) is a method of statistical analysis of multivariate variables. It assesses the regularity and variability among samples through certain recombined composite variables, and can express as much information as possible about the original variables by downscaling these new variables [22,23,24]. In the high-quality principal component analysis model. There are two principal components, PC1 and PC2, with an overall cumulative contribution of approximately 60% or even higher [24]. The principal component analysis of chrysanthemum and wild chrysanthemum samples in this study is shown in the PAC score plot.

**Figure 5:**
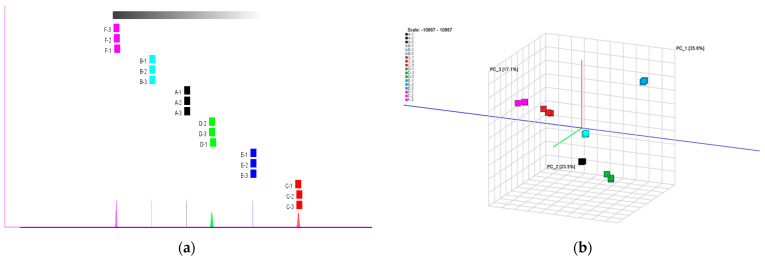
(**a**) Fingerprint similarity based on the Euclidean distance of different samples; (**b**) results of the PCA analysis of six samples.

As shown in Figure 5a, PC1 is 35.6% and PC2 is 23.5%, for a total cumulative contribution of 59.1%. As can be seen from Figure 5b, both samples A and D are located in the negative region of PC2 and the positive region of PC1, and both samples C and F are located in the positive region of PC2 and the negative region of PC1, which are in the same region but in different positions, and can be seen that there are more significant differences between them. The positions of B, D, E, and F are similar to the four quadrants of the coordinate axis, A is similar to the negative half-axis of the vertical axis, and the positions of B and C are in the negative half-axis and positive half-axis of the vertical axis, respectively, and there is a clear distinction between the positive and negative regions. The results showed that there were large differences in volatile constituent substances between chrysanthemums of different origins and significant differences between chrysanthemums and wild chrysanthemums. In a similar study, the flavor components of asparagus were clearly identified by analyzing the major volatile components in asparagus from different origins and different harvesting periods and the relationship between them, and significant differences between the volatile organic compounds of different asparagus were determined by combining and comparing the PCA analysis with the other two analyses [25].

Table 1 represents the Euclidean distance values between the five chrysanthemum samples and the wild chrysanthemum. From the results of the Euclidean distance analysis, it can be seen that the distances between chrysanthemum samples of different origins can be clearly distinguished. The average Euclidean distance between A and B is 14,200,000; between A and D is 10,388,888.889; between D and E is 16,566,666.667; and between E and C is 18,900,000. Therefore, E and C are the furthest away, i.e., the difference between Hang-chrysanthemum and Chu-chrysanthemum have the greatest difference. Sample E was located in the positive zone of PC2 and the positive zone of PC1, which was in a different zone from the rest of the samples, and it could be more clearly distinguished between chrysanthemums and wild chrysanthemums of different origins. However, principal component analysis failed to identify specific volatile organic compounds, and the relationship between the relevant components and treatments has not been clarified.

The combination of the HS-GC-IMS technique and PCA can visualize, quickly and accurately differentiate between differentiated substances and can determine the relationship between their main components and treatments [26]. In a similar study, sensory evaluation and principal component analysis of chrysanthemum teas from Anhui, Hunan and Hangzhou using e-tongue and PCA demonstrated that chrysanthemum teas from different origins had significant differences, especially in bitter and astringent flavors [27]. Another study examined volatile flavor compounds in the leaves of 15 taxa of Korean native chrysanthemum species and identified 45 volatile organic compounds using GC-MS [28].

### 2.4. Qualitative Analysis of Volatile Components Profiles of Chrysanthemum and Wild Chrysanthemum Samples from Different Origins

Currently, 41 volatile components of floral aroma have been identified in Chrysanthemum, mainly including terpenoids and hydrocarbons, of which terpenoids accounted for 11–54% and hydrocarbons accounted for 10–59% [8,29]. Wang et al. [30] utilized HS-LPME-GC to determine the volatile components in dried chrysanthemums and identified several representative components, including cineole, camphor, borneol and caryophyllene.

**Figure 6:**
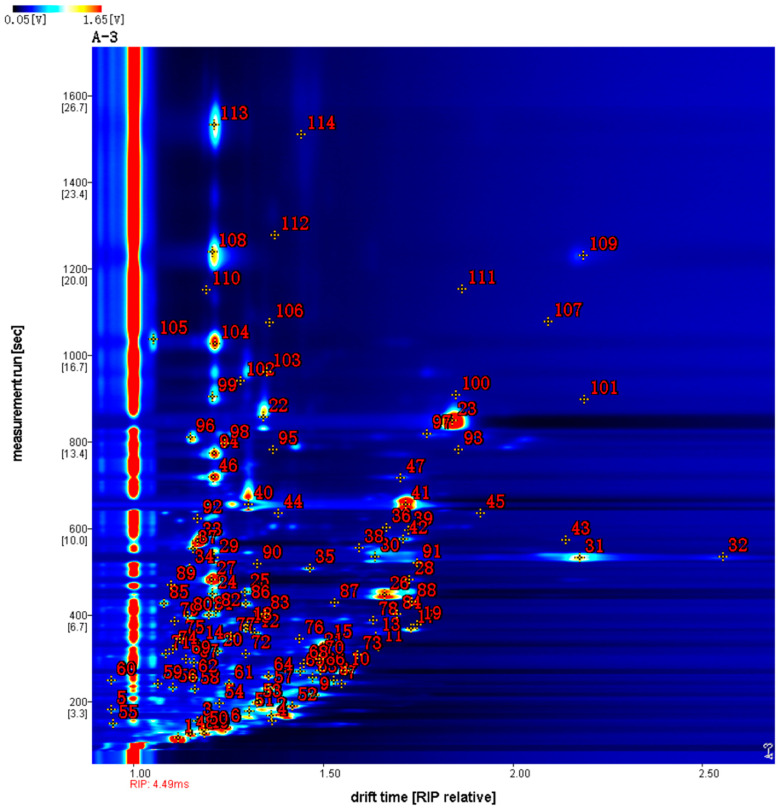
Qualitative characterization information for samples.

In the present study, volatile organic compounds from chrysanthemums and wild chrysanthemums of different origins were analyzed using HS-GC-IMS, and the qualitative characterization information expressed is shown in Figure 6, which has the differential time as the horizontal coordinate, the resolving time as the vertical coordinate, and the numbers in red represent the number of detected peaks. A total of 114 peaks were detected and 60 compounds were identified, including 14 alcohols, 12 esters, 11 hydrocarbons, 9 ketones, 7 aldehydes, 3 acids, 2 camphorates, 1 pyrazine and 1 furan. Of 2-methyl-1-propanol, 1-hexanol, 2-hexenal, 2-methybutyl acetate, 3-methylbutyl acetate, 3-methylbutanoic acid, camphor, alpha-pinene, camphene, beta-pinene, benzaldehyde, 1-octen-3-o l, cymene, hexylacetate, gamma-terpinene, (E)-2-pentenal, 3-hexanone, 2-methyl-1-pentanol, heptanal, gamma-butyrolactone, citronellal, bornyl acetate, 22 compounds were found to be available in monomeric and dimeric forms, respectively. Of these, since some compounds migrated at different times, it is presumed that their monomers underwent a polymerization reaction to form a polymer. Migration time is often a parameter used to determine the size and shape of molecules in biochemical analysis, especially in electrophoretic techniques. Dimers and multimers will have different migration times because their molecular weights and shapes affect their migration rates during electrophoresis. For example, four compounds, myrcene, beta-piene, citronellal, alpha-pinene, have a multimeric form. Compared with previous studies, the present study identified the most diverse VOCs in Chrysanthemum and wild chrysanthemum. The specific compound information is shown in Table 2.

There are significant differences in the physical properties of the different classes of compounds, which are especially evident in the odor. The flavor will vary somewhat depending on the class and content of the compounds contained. From Table 2, it can be found that both chrysanthemum and wild chrysanthemum have a cool, pungent and strong medicinal odor, which mainly comes from the camphor-like substances contained in them. Combined with Table 3, it can be found that the chrysanthemum has more p-cymene, so it will give off a citrus flavor and wood aroma; the content of 2,4-octadienal in chrysanthemum is much higher than that of other chrysanthemums, so it has a more significant melon and pear aroma; and in wild chrysanthemums, it contains a higher amount of alpha-pinene, citronellal and alpha-thujone, with the alpha-pinene odor described as terpene, peppermint, and pine, the citronellal odor as lemon, lemongrass, and rose, and the alpha-thujone odor as cypress and menthol. These substances may be the main reason why wild chrysanthemums have a minty odor.

In summary, it can be seen that alcohols accounted for 23.3% and esters for 20% of the volatile components identified. Through relevant database queries, the 2-methyl-1-propanol odor was described as fresh, boozy and leathery, and the 1-hexanol odor was described as fresh, fruity, alcoholic, sweet and green. Gamma-butyrolactone odor was described as creamy, fatty and caramel, camphene odor was described as fruity sweet, hexyl acetate odor was described as fruity, greenish, apple, banana and sweet, 3-methylbutyl acetate odor was described as sweet, banana fruity, ethyl 2-ethylpropanoate odor was described as sweet, fruity, alcoholic and rum. Methyl propanoate odor was described as sweet, fruity, alcoholic and rum [31]. It has been hypothesized that the alcoholic flavor and sweetness imparted by chrysanthemums and wild chrysanthemums may be related to the alcohol and ester components 2-methyl-1-propanol, 2-methylbutanol, 1-hexanol, gamma-butyrolactone, camphene, hexyl acetate, 3-methylbutyl acetate and ethyl 2-methylpropanoate which are alcohol and ester components related.

### 2.5. Hierarchical Cluster Analysis Heat Map

To further analyze the differences in VOCs in chrysanthemum samples from different origins, hierarchical cluster analysis (HCA) thermograms were generated. HCA can be used to distinguish between different sample clusters so that the degree of variation in the composition of the test samples can be clearly seen [32].

**Figure 7:**
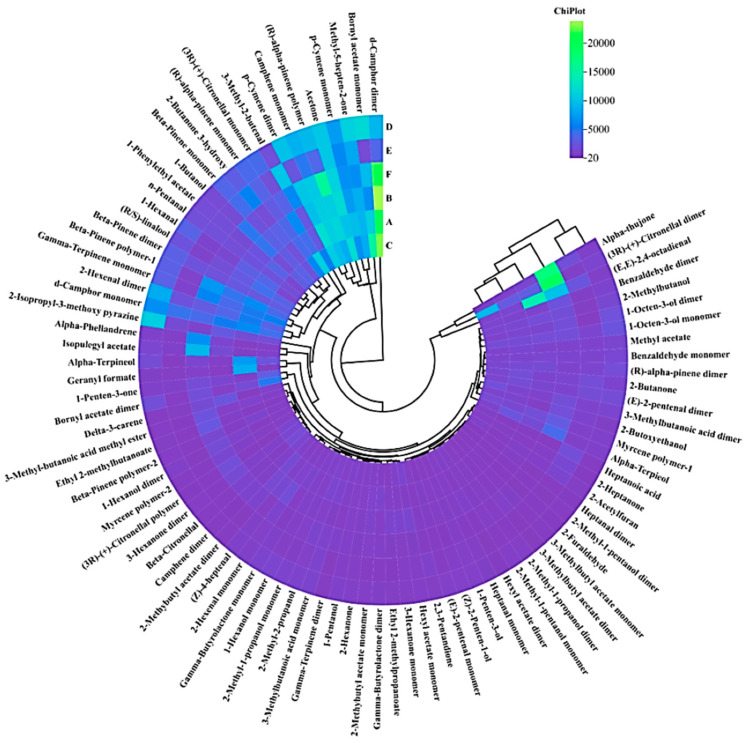
HCA of volatile components in six samples.

**Figure 8:**
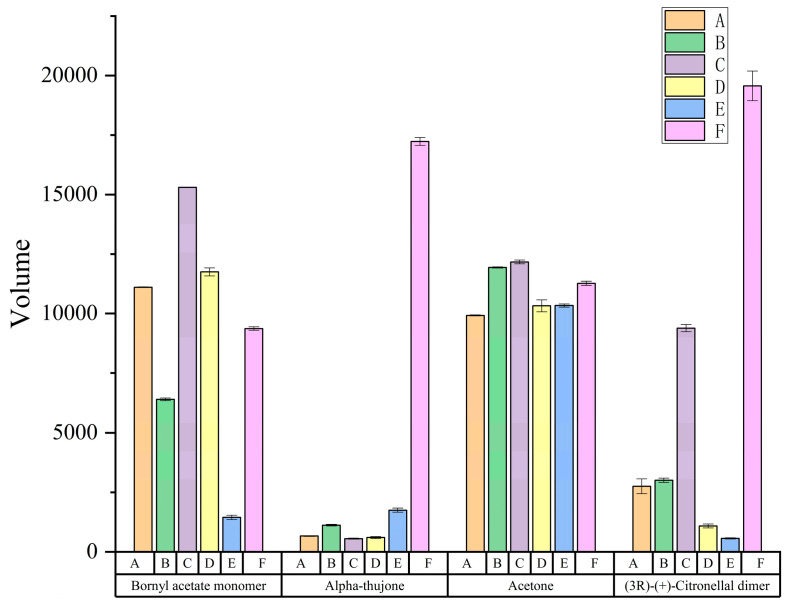
Representative component difference chart.

It is clear from Figure 7 that the relative content of volatiles varies between different chrysanthemum samples. Among the chrysanthemums of different origins, the volatile substance content in the sample of C (Chu-chrysanthemum) was higher and more diversified, and the volatile substance content in E (Hang-chrysanthemum) was lower than that in chrysanthemums of other origins. As can be seen from the graph, bornyl acetate monomer and Acetone are the most abundant in C. For F (wild chrysanthemum), it had significantly higher volatiles and higher species abundance than chrysanthemum, with higher levels of citronellal dimer, alpha-pinene polymer and alpha-phellandrene than chrysanthemum. The specific differences are shown in Figure 8, which can more clearly see that alpha-phellandrene in wild chrysanthemum is much higher than that in chrysanthemum. And combined with the analysis of its qualitative results, the compositional differences between chrysanthemums and wild chrysanthemums of different origins can be more clearly visualized. In this study, HS-GC-IMS was used to analyze the VOCs in chrysanthemum samples and wild chrysanthemum from different origins with fast response and high sensitivity.

## 3. Materials and Methods

### 3.1. Sample Preparation

**Figure 9:**
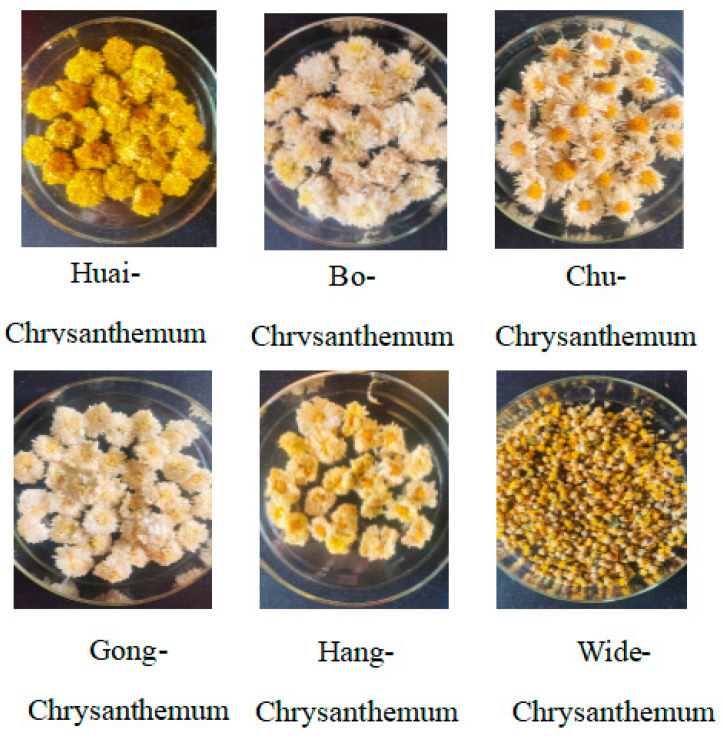
Chrysanthemum and wild chrysanthemum appearance.

All chrysanthemum and wild chrysanthemum samples were obtained from the source and were dried products. The representative appearance of these products is shown in Figure 9. Huai-chrysanthemum from Jiaozuo, Henan Province, Bo-chrysanthemum from Bozhou, Anhui Province, Chu-chrysanthemum from Chuzhou, Anhui Province, Gong-chrysanthemum from Huangshan, Anhui Province, Hang-chrysanthemum from Hangzhou, Zhejiang Province, and wild chrysanthemum from Dabie Mountain were pulverized by using a pulverizer, and subsequently sifted through an 80-mesh sieve, and their powders were obtained in order, and were named A, B, C, D, E, and F. The powder was then sifted through an 80-mesh sieve, and then sieved into the powder.

### 3.2. The HS-GC-MS System

Six samples were analyzed by FlavourSpec^®^ gas-phase ion mobility spectrometry (the G.A.S. Department of Shandong Hai Neng Science Instrument Co., Ltd., Jinan, China). 2.0 g of each sample was accurately weighed and placed in 20 mL headspace flasks and incubated at 80 °C and 500 r/min for 15 min, then the samples were injected with a sample volume of 500 µL and a syringe temperature of 85 °C. The samples were then analyzed by FlavourSpec^®^ gas-phase ion mobility spectrometry (the G.A.S. Department of Shandong Hai Neng Science Instrument Co., Ltd., Jinan, China). The samples were then analyzed.

The gas chromatographic column was a MXT-5 (the G.A.S. Department of Shandong Hai Neng Science Instrument Co., Ltd., Jinan, China) capillary column (15 m × 0.53 mm × 1 μm) at 60 °C, and the carrier gas was high-purity nitrogen (purity ≥ 99.999%); the program was boosted with the initial flow rate of 2.0 mL/min for 2 min, and then the flow rate was linearly increased to 10.0 mL/min within 8 min, and then linearly increased to 100.0 mL/min within 10 min. 100.0 mL/min within 10 min. Samples were injected through a CTC-PAL 3 static headspace autosampler (the G.A.S. Department of Shandong Hai Neng Science Instrument Co., Ltd., Jinan, China), and the temperature of the injection port was guaranteed to be 80 °C. The operation was stopped after 20 min.

Then, the ion mobility mass spectrometry (IMS) separation and detection were carried out. The ionization source was a tritium source (3H); the length of the migration tube was 53 mm; the electric field strength was 500 V/cm; the temperature of the migration tube was 45 °C; the drift gas was high-purity nitrogen (purity ≥ 99.999%); the flow rate was set at 150 mL/min; and the positive ion mode was used to carry out the measurements in three sets of parallel for each sample.

### 3.3. Data Analysis

The calibration curves of retention time and retention index were established by GC-IMS library search software (version 1.0.3) and Laboratory Analytical Viewer (LAV), and then the retention index of the target was calculated from the retention time of the target, and then searched and compared with the GC retention index (NIST 2020) database and IMS migration time database built in the VOCal software (NIST 2020).The target was then characterized by searching and comparing the GC retention index database (NIST 2020) and the IMS migration time database built in VOCal software (NIST 2020).

Reporter, Gallery Plot and Dynamic PCA plug-ins in VOCal data processing software (the G.A.S. Department of Shandong Hai Neng Science Instrument Co., Ltd., Jinan, China) were utilized to generate three-dimensional spectra, two-dimensional spectra, difference spectra, fingerprints and PCA plots of volatile constituents, respectively, for the comparison of volatile organic compounds among samples.

## 4. Conclusions

Current research has confirmed that chrysanthemums are rich in antioxidant and anti-inflammatory properties, and that their effects can be maximized through consumption and medicinal use. Therefore, this paper focuses on chrysanthemums and wild chrysanthemums mentioned in the Pharmacopoeia of the People’s Republic of China (2020 edition), and analyzes the flavor substances of chrysanthemums and wild chrysanthemums of different origins involved in them. In summary, the results of this study showed that 114 peaks of volatile organic compounds (VOCs) in chrysanthemums and wild chrysanthemums from different origins were detected by HS-GC-IMS, and a total of 60 compounds including 14 alcohols, 12 esters, 11 hydrocarbons, 9 ketones, 7 aldehydes, 3 acids, 2 camphorates, 1 pyrazine, and 1 furan, were identified in this study. The following are some examples of the types of hydrocarbons. Currently, there are 26 signal peaks that have not been identified. From the results of PCA, the Euclidean distance and hierarchical cluster analysis heatmap, the use of HS-GS-IMS can completely and effectively distinguish chrysanthemums of different origins, and at the same time, more abundant volatile organic compounds were found in wild chrysanthemums, and these unknown constituents can be further characterized and determined with the help of other analytical techniques in the future.

The results of PCA analysis, cluster analysis based on the Euclidean distance and similarity analysis of fingerprints showed that chrysanthemums from different origins had their own characteristic components. The characteristic substances in Huai-chrysanthemum are 2-methyl-2-propanol, linalool, 2-butanone, 2-hexanone, geranyl formate, styralyl acetate and alpha-pinitol. Heptanal, (2E,4E)-2,4-octadienal, 3-carene, ethyl 2-methylpropanoate, Z-4-heptenal, and hexyl acetate are characteristic substances of Bo-chrysanthemum. The characteristic substances in Chu chrysanthemum are 3-hexanone, 1-penten-3-one, icicle acetate and citronellal. The characteristic substances in Gong-chrysanthemum are hexanal, pentanol, 1-octen-3-ol and 2-isopropyl-3-methoxypyrazine. Characteristic substances in Hang-chrysanthemum are ethylidene, 2,3-pentanedione, 1-penten-3-ol, 3-methyl-2-butenal, 2-acetylfuran, 2-heptanone and heptanoic acid. The characteristic substances in wild chrysanthemum are hexanol, benzaldehyde, citronellal, isoamyl acetate, methyl 3-methylbutyrate, ethyl 2-methylbutyrate and ethylene glycol monobutyl ether. And according to its chart identification, it was found that both chrysanthemum and wild chrysanthemum contain camphor-like substances, which is presumed to be the main reason why chrysanthemum and wild chrysanthemum have a strong medicinal flavor. And their volatiles are mainly alcohols and esters: 2-methyl-1-propanol, 2-methylbutanol, 1-hexanol, gamma-butyrolactone, camphene, hexyl acetate, 3-methylbutyl acetate, and ethyl 2-methylpropanoate, which may also be the main reason for their boozy and sweet flavor.

In conclusion, by characterizing the volatile substances and using the fingerprints obtained, the differences in the volatile organic compounds of chrysanthemums and wild chrysanthemums were analyzed with a view to providing a theoretical basis for the development of chrysanthemums and wild chrysanthemums in the research and the development of chrysanthemums and wild chrysanthemums in the study and development of food flavors or functional food flavors.

## Figures and Tables

**Table 1:** Euclidean distance values between different samples.

	A-1	A-2	A-3	B-1	B-2	B-3	C-1	C-2	C-3	D-1	D-2	D-3	E-1	E-2	E-3	F-1	F-2	F-3
A-1	0	43,157.97	109,721.8	1.38 × 10^7^	1.40 × 10^7^	1.42 × 10^7^	1.30 × 10^7^	1.40 × 10^7^	1.51 × 10^7^	1.08 × 10^7^	1.01 × 10^7^	1.02 × 10^7^	1.88 × 10^7^	1.86 × 10^7^	1.83 × 10^7^	1.92 × 10^7^	1.89 × 10^7^	2.10 × 10^7^
A-2	43,157.97	0	29,902.04	1.40 × 10^7^	1.42 × 10^7^	1.44 × 10^7^	1.30 × 10^7^	1.39 × 10^7^	1.50 × 10^7^	1.08 × 10^7^	1.01 × 10^7^	1.02 × 10^7^	1.88 × 10^7^	1.86 × 10^7^	1.83 × 10^7^	1.90 × 10^7^	1.88 × 10^7^	2.09 × 10^7^
A-3	109,721.8	29,902.04	0	1.42 × 10^7^	1.44 × 10^7^	1.46 × 10^7^	1.32 × 10^7^	1.41 × 10^7^	1.51 × 10^7^	1.08 × 10^7^	1.02 × 10^7^	1.03 × 10^7^	1.90 × 10^7^	1.88 × 10^7^	1.85 × 10^7^	1.92 × 10^7^	1.89 × 10^7^	2.10 × 10^7^
B-1	1.38 × 10^7^	1.40 × 10^7^	1.42 × 10^7^	0	10,937.91	29,639.99	1.63 × 10^7^	1.72 × 10^7^	1.83 × 10^7^	1.84 × 10^7^	1.80 × 10^7^	1.82 × 10^7^	2.39 × 10^7^	2.37 × 10^7^	2.34 × 10^7^	1.44 × 10^7^	1.45 × 10^7^	1.60 × 10^7^
B-2	1.40 × 10^7^	1.42 × 10^7^	1.44 × 10^7^	10,937.91	0	15,802.00	1.63 × 10^7^	1.73 × 10^7^	1.84 × 10^7^	1.86 × 10^7^	1.82 × 10^7^	1.83 × 10^7^	2.42 × 10^7^	2.41 × 10^7^	2.37 × 10^7^	1.45 × 10^7^	1.46 × 10^7^	1.61 × 10^7^
B-3	1.42 × 10^7^	1.44 × 10^7^	1.46 × 10^7^	29,639.99	15,802.00	0	1.64 × 10^7^	1.73 × 10^7^	1.83 × 10^7^	1.87 × 10^7^	1.84 × 10^7^	1.85 × 10^7^	2.46 × 10^7^	2.44 × 10^7^	2.41 × 10^7^	1.44 × 10^7^	1.46 × 10^7^	1.60 × 10^7^
C-1	1.30 × 107	1.30 × 10^7^	1.32 × 10^7^	1.63 × 10^7^	1.63 × 10^7^	1.64 × 10^7^	0	563,573.6	850,431.2	1.72 × 10^7^	1.73 × 10^7^	1.75 × 10^7^	1.88 × 10^7^	1.86 × 10^7^	1.83 × 10^7^	1.68 × 10^7^	1.66 × 10^7^	1.81 × 10^7^
C-2	1.40 × 10^7^	1.39 × 10^7^	1.41 × 10^7^	1.72 × 10^7^	1.73 × 10^7^	1.73 × 10^7^	563,573.6	0	140,683.2	1.69 × 10^7^	1.79 × 10^7^	1.82 × 10^7^	1.95 × 10^7^	1.93 × 10^7^	1.93 × 10^7^	1.74 × 10^7^	1.66 × 10^7^	1.80 × 10^7^
C-3	1.51 × 10^7^	1.50 × 10^7^	1.51 × 10^7^	1.83 × 10^7^	1.84 × 10^7^	1.83 × 10^7^	850,431.2	140,683.2	0	1.80 × 10^7^	1.92 × 10^7^	1.95 × 10^7^	2.06 × 10^7^	2.04 × 10^7^	2.03 × 10^7^	1.75 × 10^7^	1.67 × 10^7^	1.80 × 10^7^
D-1	1.08 × 10^7^	1.08 × 10^7^	1.08 × 10^7^	1.84 × 10^7^	1.86 × 10^7^	1.87 × 10^7^	1.72 × 10^7^	1.69 × 10^7^	1.80 × 10^7^	0	487,389.5	506,111.1	1.64 × 10^7^	1.65 × 10^7^	1.67 × 10^7^	2.50 × 10^7^	2.37 × 10^7^	2.66 × 10^7^
D-2	1.01 × 10^7^	1.01 × 10^7^	1.02 × 10^7^	1.80 × 10^7^	1.82 × 10^7^	1.84 × 10^7^	1.73 × 10^7^	1.79 × 10^7^	1.92 × 10^7^	487,389.5	0	23,838.09	1.65 × 10^7^	1.65 × 10^7^	1.64 × 10^7^	2.51 × 10^7^	2.43 × 10^7^	2.73 × 10^7^
D-3	1.02 × 10^7^	1.02 × 10^7^	1.03 × 10^7^	1.82 × 10^7^	1.83 × 10^7^	1.85 × 10^7^	1.75 × 10^7^	1.82 × 10^7^	1.95 × 10^7^	506,111.1	23,838.09	0	1.67 × 10^7^	1.67 × 10^7^	1.67 × 10^7^	2.52 × 10^7^	2.44 × 10^7^	2.73 × 10^7^
E-1	1.88 × 10^7^	1.88 × 10^7^	1.90 × 10^7^	2.39 × 10^7^	2.42 × 10^7^	2.46 × 10^7^	1.88 × 10^7^	1.95 × 10^7^	2.06 × 10^7^	1.64 × 10^7^	1.65 × 10^7^	1.67 × 10^7^	0	33,015.81	145,579.7	2.88 × 10^7^	2.80 × 10^7^	3.16 × 10^7^
E-2	1.86 × 10^7^	1.86 × 10^7^	1.88 × 10^7^	2.37 × 10^7^	2.41 × 10^7^	2.44 × 10^7^	1.86 × 10^7^	1.93 × 10^7^	2.04 × 10^7^	1.65 × 10^7^	1.65 × 10^7^	1.67 × 10^7^	33,015.81	0	55,887.57	2.85 × 10^7^	2.77 × 10^7^	3.13 × 10^7^
E-3	1.83 × 10^7^	1.83 × 10^7^	1.85 × 10^7^	2.34 × 10^7^	2.37 × 10^7^	2.41 × 10^7^	1.83 × 10^7^	1.93 × 10^7^	2.03 × 10^7^	1.67 × 10^7^	1.64 × 10^7^	1.67 × 10^7^	145,579.7	55,887.57	0	2.81 × 10^7^	2.75 × 10^7^	3.10 × 10^7^
F-1	1.92 × 10^7^	1.90 × 10^7^	1.92 × 10^7^	1.44 × 10^7^	1.45 × 10^7^	1.44 × 10^7^	1.68 × 10^7^	1.74 × 10^7^	1.75 × 10^7^	2.50 × 10^7^	2.51 × 10^7^	2.52 × 10^7^	2.88 × 10^7^	2.85 × 10^7^	2.81 × 10^7^	0	202,443.5	401,301.5
F-2	1.89 × 10^7^	1.88 × 10^7^	1.89 × 10^7^	1.45 × 10^7^	1.46 × 10^7^	1.46 × 10^7^	1.66 × 10^7^	1.66 × 10^7^	1.67 × 10^7^	2.37 × 10^7^	2.43 × 10^7^	2.44 × 10^7^	2.80 × 10^7^	2.77 × 10^7^	2.75 × 10^7^	202,443.5	0	268,847.8
F-3	2.10 × 10^7^	2.09 × 10^7^	2.10 × 10^7^	1.60 × 10^7^	1.61 × 10^7^	1.60 × 10^7^	1.81 × 10^7^	1.80 × 10^7^	1.80 × 10^7^	2.66 × 10^7^	2.73 × 10^7^	2.73 × 10^7^	3.16 × 10^7^	3.13 × 10^7^	3.10 × 10^7^	401,301.5	268,847.8	0

**Table 2:** Results of the qualitative analysis of six samples (odor description queried at: https://www.femaflavor.org/ (accessed on 10 August 2024)).

Category	No.	Compound	CAS#	MW	(RI)	(Rt [min])	(Dt [a. u.])	Odor
Alcohols	**2**	1-Butanol	71-36-3	74.1	655.9	2.797	1.36641	wine
	**3**	2-Methyl-1-propanol ^a^	78-83-1	74.1	625.5	2.583	1.17021	fresh, alcoholic, leather
	**4**	2-Methyl-1-propanol ^b^	78-83-1	74.1	623.8	2.571	1.36774	ethereal, green, tropical fruity
	**5**	1-Penten-3-ol	616-25-1	86.1	683.4	3.005	0.94351	
	**9**	2-Methylbutanol	137-32-6	88.1	732.5	3.580	1.48024	roast onion, fruity, floral, wine
	**12**	1-Hexanol ^a^	111-27-3	102.2	874.8	5.990	1.32128	fresh, fruity, wine, sweet, green
	**13**	1-Hexanol ^b^	111-27-3	102.2	868.7	5.859	1.64064	mushroom, lavender, rose, hay
	**37**	1-Octen-3-ol ^a^	3391-86-4	128.2	987.1	9.219	1.15932	
	**38**	1-Octen-3-ol ^b^	3391-86-4	128.2	987.8	9.246	1.59633	camphor
	**48**	2-Methyl-2-propanol	75-65-0	74.1	538.2	2.060	1.15207	
	**60**	(Z)-2-Penten-1-ol	1576-95-0	86.1	772.1	4.142	0.94409	green, plastic, rubber
	**61**	1-Pentanol	71-41-0	88.1	762.9	4.003	1.25345	balsamic
	**72**	2-Methyl-1-pentanol ^a^	105-30-6	102.2	833.1	5.159	1.29837	pungent
	**73**	2-Methyl-1-pentanol ^b^	105-30-6	102.2	831.3	5.125	1.59325	
	**94**	Linalool	78-70-6	154.3	1101.2	12.899	1.21493	thujone, menthol lemon, lemongrass, rose
	**102**	Alpha-Terpieol	10482-56-1	154.3	1169.5	15.678	1.28482	
	**104**	Alpha-Terpineol	98-55-5	154.3	1200.4	17.128	1.21709	pine terpenoid, citrus, floral green, leaf, rose
	**113**	Geranyl formate	105-86-2	182.3	1340.4	25.552	1.21552	
Aldehydes	**10**	1-Hexanal	66-25-1	100.2	796.2	4.519	1.56044	fresh, green, fat, fruity bitter almond, cherry, nutty
	**34**	Benzaldehyde ^a^	100-52-7	106.1	965.4	8.474	1.15018	
	**35**	Benzaldehyde ^b^	100-52-7	106.1	965.1	8.463	1.46836	potato, peas
	**56**	(E)-2-Pentenal ^a^	1576-87-0	84.1	753.8	3.872	1.10677	
	**64**	3-Methyl-2-butenal	107-86-8	84.1	783.7	4.322	1.35879	Fruity sweet, woody, almond, bready sweet, caramel, nutty, tobacco
	**71**	2-Furaldehyde	98-01-1	96.1	833.1	5.159	1.0866	
	**80**	(Z)-4-Heptenal	6728-31-0	112.2	902.2	6.627	1.14558	
	**83**	Heptanal ^a^	111-71-7	114.2	904.6	6.690	1.34951	pungent, ether aldehyde, fatty, green herbs,
	**84**	Heptanal ^b^	111-71-7	114.2	905.4	6.709	1.69633	
Acids	**20**	3-Methylbutanoic acid ^a^	503-74-2	102.1	839.4	5.276	1.22389	sour, foot sweat, cheese citrus, rose, woody, blueberry floral, lilac, terpene
	**21**	3-Methylbutanoic acid ^b^	503-74-2	102.1	839.4	5.276	1.49027	
	**95**	Heptanoic acid	111-14-8	130.2	1104.3	13.015	1.36945	
	**103**	Beta-citronellal	106-23-0	154.3	1176.8	16.011	1.35488	
Hydrocarbon	**14**	2-Hexenal ^a^	505-57-7	98.1	852.2	5.524	1.1752	sweet almonds, fruity, leaves, apples, plums, vegetables Terpene, Mint, Pine
	**15**	2-Hexenal ^b^	505-57-7	98.1	854.4	5.568	1.51461	
	**24**	Alpha-pinene ^a^	7785-70-8	136.2	932.8	7.464	1.2099	
	**25**	Alpha-pinene ^b^	7785-70-8	136.2	935.7	7.548	1.29564	
	**29**	Beta-pinene ^a^	127-91-3	136.2	977	8.866	1.2148	resin, green
	**30**	Beta-pinene ^b^	127-91-3	136.2	978.7	8.924	1.63805	
	**36**	Alpha-phellandrene	99-83-2	136.2	1013.5	10.039	1.67	dill
	**39**	Delta-3-carene	13466-78-9	136.2	1009.7	9.931	1.72659	citrus, lemon, woody fresh, citrus, terpene,
	**40**	p-Cymene ^a^	99-87-6	134.2	1043	10.924	1.30429	
	**41**	p-Cymene ^b^	99-87-6	134.2	1042.5	10.906	1.72029	woody, spice oil, wood, terpenes, lemon,
	**46**	Gamma-terpinene ^a^	99-85-4	136.2	1075.1	11.972	1.21183	
	**47**	Gamma-terpinene ^b^	99-85-4	136.2	1074.4	11.949	1.70654	lime, herbs green grassy, faint banana
	**52**	n-Pentanal	110-62-3	86.1	698.7	3.162	1.42093	
	**57**	(E)-2-pentenal ^b^	1576-87-0	84.1	751.9	3.844	1.35879	potato, peas fatty, sour, sweat
	**97**	(E,E)-2,4-Octadienal	30361-28-5	124.2	1120.4	13.628	1.77684	
	**99**	Citronellal ^a^	2385-77-5	154.3	1155.4	15.060	1.21008	mung bean
	**100**	Citronellal ^b^	2385-77-5	154.3	1157.6	15.155	1.85234	lemon, lemongrass, rose
Ketones	**51**	1-Penten-3-one	1629-58-9	84.1	674.9	2.939	1.30743	strong pungent odors
	**1**	Acetone	67-64-1	58.1	513.9	1.935	1.11983	fresh, apple, pear
	**6**	2-Butanone	78-93-3	72.1	584.8	2.323	1.24445	fruity, camphor
	**33**	6-Methyl-5-hepten-2-one	110-93-0	126.2	994.9	9.505	1.1714	citrus, fruity, moldy, ketone
	**53**	2-Butanone 3-hydroxy	513-86-0	88.1	710	3.296	1.3279	butter, cream cream, caramel, nuts, cheese
	**54**	2,3-Pentandione	600-14-6	100.1	706.7	3.256	1.22835	
	**62**	3-Hexanone ^a^	589-38-8	100.2	778	4.232	1.16011	fruity, grape, sweet, rum
	**63**	3-Hexanone ^b^	589-38-8	100.2	778	4.232	1.47481	
	**66**	2-Hexanone	591-78-6	100.2	795.9	4.516	1.49481	fruity, fungal, meaty, buttery fruity, slight medicinal fragrance
	**78**	2-Heptanone	110-43-0	114.2	895	6.443	1.63381	
	**93**	Alpha-thujone	546-80-5	152.2	1104.9	13.038	1.86	cream, fat, caramel
Esters	**7**	Ethyl 2-methylpropanoate	97-62-1	116.2	763.3	4.010	1.55185	sweet, fruity, alcoholic, rummy
	**8**	3-Methyl-butanoic acid methyl ester	556-24-1	116.2	772.6	4.148	1.53037	strong apple, pineapple
	**11**	Ethyl 2-methylbutanoate	7452-79-1	130.2	847	5.422	1.64923	Apple fruity
	**16**	2-Methybutyl acetate ^a^	624-41-9	130.2	881.5	6.135	1.29836	
	**17**	2-Methybutyl acetate ^b^	624-41-9	130.2	881.5	6.135	1.73373	sweet, banana, fruity
	**18**	3-Methylbutyl acetate ^a^	123-92-2	130.2	886.5	6.245	1.30409	
	**19**	3-Methylbutyl acetate ^b^	123-92-2	130.2	888.7	6.295	1.75091	
	**44**	Hexyl acetate ^a^	142-92-7	144.2	1032	10.584	1.38451	fruity, green, apple, banana, sweet
	**45**	Hexyl acetate ^b^	142-92-7	144.2	1031.2	10.561	1.91656	
	**49**	Methyl acetate	79-20-9	74.1	544.1	2.091	1.19021	Ethereal cream, fat, caramel
	**85**	Gamma-butyrolactone ^a^	96-48-0	86.1	920.2	7.108	1.08306	
	**86**	Gamma-butyrolactone ^b^	96-48-0	86.1	919.5	7.089	1.2989	gardenia
	**105**	1-Phenylethyl acetate	93-92-5	164.2	1203.3	17.270	1.05594	
	**108**	Bornyl acetate ^a^	76-49-3	196.3	1265.9	20.653	1.20966	herbal, pine leaf
	**109**	Bornyl acetate ^b^	76-49-3	196.3	1263.7	20.525	2.18845	
	**112**	Isopulegyl acetate	89-49-6	196.3	1276.6	21.292	1.37376	mint
Pyrazine	**98**	2-Isopropyl-3-methoxy pyrazine	25773-40-4	152.2	1110.9	13.261	1.24178	fatty, green, pear, melon
Polymers	**26**	Alpha-pinene ^c^	7785-70-8	136.2	932.5	7.457	1.66557	Terpene, Mint, Pine
	**42**	Myrcene ^c^-1	123-35-3	136.2	996.9	9.575	1.71354	must, spice, balsamic lemon, lemongrass, rose resin, green
	**43**	Myrcene ^c^-2	123-35-3	136.2	996.2	9.552	2.14292	
	**101**	Citronellal ^c^	2385-77-5	154.3	1153.2	14.965	2.19099	
	**31**	Beta-pinene ^c^-1	127-91-3	136.2	977.4	8.880	2.17894	
	**32**	Beta-pinene ^c^-2	127-91-3	136.2	978.1	8.902	2.55809	
Furan	**79**	2-Acetylfuran	1192-62-7	110.1	894.5	6.431	1.11134	pear, banana, fruity, slight medicinal fragrance
Camphor	**22**	Camphor ^a^	464-49-3	152.2	1136.6	14.274	1.34427	cool, pungent, strong medicinal taste
	**23**	Camphor ^b^	464-49-3	152.2	1133.2	14.133	1.84416	
	**27**	Camphene ^a^	79-92-5	136.2	951.2	8.018	1.20745	woody, camphor
	**28**	Camphene ^b^	79-92-5	136.2	951.2	8.018	1.72926	

Note: RI, retention index; Rt, retention time; Dt, drift time. ^a^: monomer; ^b^: dimer; ^c^: polymer.

**Table 3:** Table of differences in the content of constituents between chrysanthemum and wild chrysanthemums.

Sample	A	B	C	D	E	F
Citronellal ^b^	2825.063 ± 311.767	3029.415 ± 90.208	9504.174 ± 150.258	1130.174 ± 83.035	594.981 ± 25.349	19,935.519 ± 625.756
Citronellal ^a^	4240.191 ± 152.91	6212.55 ± 134.105	10,979.493 ± 212.347	4286.946 ± 620.815	1913.36 ± 15.664	3553.182 ± 23.644
Citronellal ^c^	59.693 ± 5.994	107.97 ± 5.466	1083.988 ± 22.266	77.333 ± 9.386	68.877 ± 5.537	1807.551 ± 37.552
(E)-2-Pentenal ^b^	1544.384 ± 10.722	1012.83 ± 12.522	1023.602 ± 3.293	1541.005 ± 30.907	1500.362 ± 59.313	1024.219 ± 50.792
(E)-2-Pentenal ^a^	396.835 ± 1.324	335.796 ± 3.928	100.316 ± 3.366	535.949 ± 14.15	203.75 ± 4.235	177.514 ± 2.932
(E,E)-2,4-Octadienal	1192.651 ± 26.982	14,708.422 ± 63.505	898.056 ± 63.246	556.223 ± 12.68	2338.393 ± 114.052	9917.439 ± 148.389
Alpha-pinene ^b^	1402.402 ± 3.498	1371.06 ± 9.938	1166.338 ± 26.807	1633.6 ± 31.423	1079.45 ± 6.869	791.856 ± 16.939
Alpha-pinene ^a^	4071.159 ± 11.178	3786.45 ± 29.14	5877.256 ± 40.282	3940.424 ± 25.801	3500.888 ± 62.541	2875.96 ± 55.359
Alpha-pinene ^c^	11,442.659 ± 111.613	10,791.332 ± 34.41	8366.991 ± 195.356	9223.346 ± 60.514	3766.755 ± 121.371	16,081.79 ± 400.241
Linalool	4402.249 ± 34.666	2754.093 ± 21.022	3484.353 ± 83.691	2132.124 ± 79.75	787.952 ± 18.909	1691.72 ± 53.114
(Z)-2-Penten-1-ol	316.707 ± 3.598	287.878 ± 6.144	191.349 ± 5.996	517.487 ± 14.231	367.465 ± 2.067	108.935 ± 5.568
(Z)-4-Heptenal	635.594 ± 33.284	1245.405 ± 6.179	273.128 ± 4.053	610.033 ± 27.153	546.022 ± 29.61	277.641 ± 21.204
1-Butanol	1715.981 ± 12.401	4728.318 ± 35.54	3075.394 ± 45.504	1393.119 ± 37.33	1574.953 ± 63.172	4988.075 ± 194.39
1-Hexanal	3724.348 ± 37.997	3130.357 ± 62.556	1158.475 ± 295.215	3339.963 ± 341.334	918.021 ± 111.395	1249.278 ± 106.966
1-Hexanol ^b^	196.601 ± 8.151	788.773 ± 16.713	283.705 ± 4.958	434.067 ± 7.799	59.811 ± 1.873	1584.674 ± 81.02
1-Hexanol ^a^	644.557 ± 5.216	616.344 ± 10.039	574.513 ± 6.98	1036.935 ± 23.295	318.547 ± 7.136	463.043 ± 14.268
1-Octen-3-ol ^b^	1053.852 ± 20.27	1000.329 ± 17.311	851.294 ± 27.08	2118.039 ± 24.059	202.776 ± 12.17	1785.737 ± 31.121
1-Octen-3-ol ^a^	1369.518 ± 9.325	1615.156 ± 5.563	1554.763 ± 4.213	2254.018 ± 44.902	1096.426 ± 89.617	1737.718 ± 109.048
1-Pentanol	538.727 ± 16.025	541.481 ± 4.803	454.079 ± 10.98	647.792 ± 17.66	358.871 ± 1.603	461.167 ± 5.109
1-Penten-3-ol	300.365 ± 7.087	367.502 ± 8.734	182.029 ± 11.154	519.26 ± 15.326	503.944 ± 6.752	274.31 ± 8.462
1-Penten-3-one	554.95 ± 4.08	1035.61 ± 8.517	3173.32 ± 80.688	623.15 ± 26.815	584.78 ± 7.582	1668.652 ± 128.461
1-Phenylethyl acetate	3392.28 ± 23.211	2290.869 ± 8.987	1352.088 ± 35.373	1584.211 ± 4.707	1751.044 ± 37.727	1370.581 ± 23.111
2,3-Pentandione	159.694 ± 6.152	334.943 ± 1.868	254.51 ± 5.717	279.47 ± 7.116	647.531 ± 3.991	182.076 ± 10.645
2-Butanone	1698.224 ± 6.728	970.721 ± 11.318	1183.41 ± 17.581	952.998 ± 47.859	1598.619 ± 43.32	1081.831 ± 18.825
2-Butanone 3-hydroxy	2055.259 ± 46.821	3284.344 ± 20.483	2123.28 ± 37.777	3107.221 ± 25.008	5591.911 ± 35.104	1650.921 ± 9.038
2-Furaldehyde	200.482 ± 2.282	72.379 ± 2.576	50.859 ± 1.601	87.573 ± 5.129	181.367 ± 3.037	42.454 ± 4.959
2-Heptanone	264.123 ± 8.224	223.118 ± 4.825	375.322 ± 5.075	99.625 ± 1.98	1343.407 ± 37.936	330.095 ± 18.373
2-Hexanone	505.35 ± 14.317	483.251 ± 3.935	455.531 ± 36.455	432.012 ± 7.946	294.856 ± 12.802	393.94 ± 3.457
2-Hexenal ^b^	5160.485 ± 51.206	3688.567 ± 31.339	3575.651 ± 39.054	8273.701 ± 52.296	2189.332 ± 35.959	2782.315 ± 52.51
2-Hexenal ^a^	743.456 ± 25.588	1753.04 ± 11.586	403.135 ± 8.843	542.754 ± 12.88	522.099 ± 10.233	806.048 ± 5.454
2-Isopropyl-3-methoxy pyrazine	1497.287 ± 11.955	2573.201 ± 42.793	1696.038 ± 45.632	10,858.284 ± 114.53	1420.291 ± 40.884	840.125 ± 22.759
2-Methybutyl acetate ^b^	1334.981 ± 17.904	1339.061 ± 7.665	685.082 ± 28.844	152.724 ± 10.614	48.278 ± 2.871	1005.383 ± 53.198
2-Methybutyl acetate ^a^	724.646 ± 6.442	756.985 ± 3.711	495.039 ± 9.392	341.205 ± 9.359	126.668 ± 1.881	522.369 ± 12.799
2-Methyl-1-pentanol ^b^	68.645 ± 3.064	163.215 ± 4.006	84 ± 4.618	73.949 ± 3.167	17.563 ± 0.43	214.544 ± 14.1
2-Methyl-1-pentanol ^a^	277.903 ± 3.554	349.819 ± 7.307	290.779 ± 5.553	351.659 ± 16.561	71.349 ± 3.495	312.84 ± 8.539
2-Methyl-1-propanol ^b^	272.323 ± 3.729	278.319 ± 5.837	267.338 ± 1.555	300.509 ± 6.006	74.069 ± 1.923	232.508 ± 13.88
2-Methyl-1-propanol ^a^	524.472 ± 16.296	632.126 ± 5.693	598.552 ± 5.415	839.38 ± 26.266	386.994 ± 13.268	556.119 ± 30.345
2-Methyl-2-propanol	1069.7 ± 7.409	541.516 ± 0.593	746.286 ± 1.98	803.986 ± 18.411	510.687 ± 11.152	940.697 ± 24.328
2-Methylbutanol	1325.873 ± 7.093	1607.571 ± 11.085	1076.535 ± 6.415	1219.632 ± 15.172	268.532 ± 7.942	1693.174 ± 51.495
3-Hexanone ^b^	606.673 ± 17.751	684.135 ± 13.451	1238.309 ± 56.229	210.639 ± 4.873	134.987 ± 0.771	1475.418 ± 75.564
3-Hexanone ^a^	412.421 ± 13.386	912.889 ± 16.892	617.404 ± 16.624	267.355 ± 8.084	311.278 ± 4.975	262.989 ± 8.16
3-Methyl-2-butenal	2614.625 ± 43.827	2484.74 ± 99.631	5757.293 ± 947.538	2023.336 ± 508.322	9940.402 ± 842.686	6269.529 ± 435.626
3-Methylbutanoic acid ^b^	954.148 ± 99.641	852.211 ± 19.269	1882.101 ± 86.377	1103.04 ± 25.177	845.929 ± 10.847	1784.733 ± 92.792
3-Methylbutanoic acid ^a^	787.781 ± 55.271	383.964 ± 14.384	505.373 ± 34.926	947.641 ± 39.816	907.718 ± 9.952	776.635 ± 9.241
3-Methylbutyl acetate ^b^	170.946 ± 8.875	164.962 ± 5.202	69.952 ± 2.655	27.028 ± 2.585	24.298 ± 4.321	582.069 ± 54.463
3-Methylbutyl acetate ^a^	233.319 ± 21.051	229.359 ± 4.58	114.067 ± 6.64	161.848 ± 10.735	62.241 ± 2.71	395.959 ± 5.334
2-Acetylfuran	137.379 ± 7.036	443.635 ± 17.858	697.362 ± 22.76	313.253 ± 3.488	1058.682 ± 37.403	369.625 ± 8.148
Acetone	9937.83 ± 23.006	11,941.912 ± 23.631	12,158.292 ± 80.719	10,257.431 ± 251.31	10,405.129 ± 65.245	11,318.299 ± 89.473
Alpha-phellandrene	253.622 ± 14.694	1073.087 ± 11.035	511.084 ± 18.506	679.988 ± 27.402	441.449 ± 10.139	6735.333 ± 192.996
Alpha-terpieol	2359.078 ± 59.81	1357.65 ± 19.441	952.215 ± 1.466	690.553 ± 11.704	645.298 ± 12.369	3967.721 ± 52.078
Alpha-terpineol	7167.358 ± 41.731	343.755 ± 8.974	2397.673 ± 98.38	1597.043 ± 83.792	175.126 ± 20.176	1067.31 ± 46.124
Alpha-thujone	654.039 ± 16.374	1123.151 ± 34.743	547.913 ± 22.677	554.919 ± 42.304	1817.227 ± 84.151	17,396.78 ± 165.955
Benzaldehyde ^b^	920.376 ± 14.175	952.486 ± 0.885	1088.496 ± 18.252	1023.338 ± 33.251	374.142 ± 8.27	2179.382 ± 46.044
Benzaldehyde ^a^	976.783 ± 10.871	1113.11 ± 6.734	952.394 ± 18.152	1333.735 ± 21.054	939.441 ± 26.309	985.417 ± 19.907
Beta-citronellal	332.559 ± 32.39	425.507 ± 16.061	1488.985 ± 51.464	195.047 ± 20.866	147.069 ± 6.58	874.15 ± 21.093
Beta-pinene ^b^	2528.678 ± 18.206	2559.66 ± 7.98	2994.272 ± 17.622	3252.752 ± 35.367	2154.97 ± 42.101	2051.414 ± 80.244
Beta-pinene ^a^	2826.033 ± 48.497	3255.064 ± 25.052	3359.369 ± 48.1	3709.292 ± 37.442	4172.802 ± 53.598	2207.279 ± 34.857
Beta-pinene ^c^-1	6196.205 ± 39.541	4718.965 ± 67.345	4100.127 ± 169.114	3302.962 ± 54.286	771.942 ± 28.491	7101.551 ± 122.436
Beta-pinene ^c^-2	943.207 ± 17.128	360.358 ± 20.508	308.354 ± 21.18	212.635 ± 9.503	116.688 ± 10.225	1263.187 ± 66.259
Bornyl acetate ^b^	1565.311 ± 14.112	501.45 ± 31.825	5115.825 ± 375.809	1812.541 ± 42.723	260.377 ± 19.161	1840.328 ± 88.389
Bornyl acetate ^a^	11,124.535 ± 16.558	6353.636 ± 59.415	15,289.999 ± 9.138	11,729.532 ± 167.038	1527.121 ± 92.602	9451.247 ± 72.889
Camphene ^b^	593.797 ± 1.099	506.467 ± 13.384	1078.246 ± 7.095	192.453 ± 15.302	88.527 ± 4.018	965.494 ± 129.185
Camphene ^a^	10,965.884 ± 33.302	10,833.183 ± 10.499	9553.585 ± 209.235	8785.027 ± 47.359	4858.39 ± 107.671	10,498.42 ± 126.534
Camphor ^b^	20,266.817 ± 138.836	23,749.914 ± 93.341	23,022.477 ± 95.772	9162.253 ± 224.637	3669.357 ± 57.587	21,994.788 ± 458.43
Camphor ^a^	6133.533 ± 103.599	5189.051 ± 30.245	4393.395 ± 51.573	6498.457 ± 59.219	4455.203 ± 75.98	3915.589 ± 17.663
Delta-3-carene	132.514 ± 9.274	2366.018 ± 10.661	669.33 ± 21.83	118.453 ± 4.696	261.679 ± 11.363	1462.493 ± 58.672
Ethyl 2-methylbutanoate	138.249 ± 8.577	489.096 ± 11.198	182.273 ± 2.251	260.743 ± 9.784	68.223 ± 3.402	2621.915 ± 169.078
Ethyl 2-methylpropanoate	318.692 ± 7.984	917.68 ± 3.255	220.792 ± 7.268	265.075 ± 7.62	78.928 ± 0.942	426.274 ± 28.982
Gamma-butyrolactone ^b^	930.888 ± 69.772	359.555 ± 7.488	354.153 ± 24.311	422.583 ± 28.419	186.657 ± 5.559	453.242 ± 8.58
Gamma-butyrolactone ^a^	909.139 ± 25.648	372.019 ± 6.16	424.385 ± 6.689	900.071 ± 25.937	572.773 ± 1.669	351.585 ± 6.624
Gamma-terpinene ^b^	556.677 ± 4.606	210.187 ± 5.344	651.832 ± 14.062	131.27 ± 2.895	38.058 ± 1.628	721.183 ± 39.372
Gamma-terpinene ^a^	5081.537 ± 40.31	3943.89 ± 51.482	5366.795 ± 63.766	3392.369 ± 56.998	858.552 ± 7.429	5846.44 ± 86.406
Geranyl formate	8583.753 ± 62.08	241.205 ± 14.802	381.155 ± 78.127	450.238 ± 75.432	231.62 ± 48.438	436.755 ± 86.56
Heptanoic acid	162.129 ± 12.607	286.251 ± 3.17	91.055 ± 2.416	109.447 ± 5.278	72.165 ± 3.461	62.626 ± 3.213
Hexyl acetate ^b^	358.53 ± 18.941	418.931 ± 4.567	315.414 ± 8.372	375.405 ± 10.132	306.776 ± 2.619	192.307 ± 11.945
Hexyl acetate ^a^	146.962 ± 8.044	142.352 ± 4.388	390.6 ± 7.076	151.54 ± 9.051	1756.449 ± 22.052	157.767 ± 5.354
Isopulegyl acetate	117.503 ± 4.234	593.248 ± 21.458	423.447 ± 14.717	79.057 ± 3.199	37.441 ± 4.691	154.621 ± 15.865
Methyl acetate	338.277 ± 12.254	1013.593 ± 3.847	967.783 ± 7.581	368.183 ± 10.435	277.988 ± 1.689	421.014 ± 12.501
Methyl-5-hepten-2-one	375.606 ± 12.349	1347.956 ± 53.871	585.792 ± 56.228	1140.322 ± 45.541	602.273 ± 64.204	9405.44 ± 157.669
Myrcene ^c^-1	1210.857 ± 10.697	925.157 ± 5.002	819.306 ± 5.759	976.111 ± 24.42	867.97 ± 7.341	887.19 ± 37.47
Myrcene ^c^-2	8948.613 ± 36.241	8451.142 ± 20.486	8279.251 ± 2.76	11,074.886 ± 48.893	7918.744 ± 80.609	6485.896 ± 50.106
n-Pentanal	1266.865 ± 16.835	1709.672 ± 8.838	1456.214 ± 38.665	633.634 ± 13.947	705.014 ± 34.15	2779.038 ± 32.353
p-Cymene ^b^	261.845 ± 11.725	506.495 ± 10.205	311.848 ± 16.235	176.002 ± 3.382	202.759 ± 3.654	1236.11 ± 67.634
p-Cymene ^a^	2142.683 ± 6.597	2304.334 ± 9.363	1738.314 ± 16.098	1788.107 ± 49.658	2005.642 ± 24.161	1753.989 ± 24.705
Heptanal ^b^	12,368.913 ± 142.87	9742.773 ± 27.733	10,713.188 ± 44.56	9971.672 ± 52.523	2469.909 ± 48.31	10,366.861 ± 179.679
Heptanal ^a^	9664.966 ± 241.851	7205.739 ± 74.51	6620.843 ± 140.607	7293.801 ± 84.535	5992.317 ± 40.622	6642.647 ± 139.93
3-Methyl-butanoic acid methyl ester	721.335 ± 8.925	549.814 ± 3.547	426.946 ± 21.264	227.048 ± 12.816	130.986 ± 3.969	2287.457 ± 119.615

Note: ^a^: monomer; ^b^: dimer; ^c^: polymer.

## Data Availability

The data are contained within this article. Samples of the compounds are not available from the authors.
